# Tumor-Targeted Gene Silencing IDO Synergizes PTT-Induced Apoptosis and Enhances Anti-tumor Immunity

**DOI:** 10.3389/fimmu.2020.00968

**Published:** 2020-06-09

**Authors:** Yujuan Zhang, Yuanyuan Feng, Yanqing Huang, Yifan Wang, Li Qiu, Yanling Liu, Shanshan Peng, Rong Li, Nanzhen Kuang, Qiaofa Shi, Yanmei Shi, Yiguo Chen, Rakesh Joshi, Zhigang Wang, Keng Yuan, Weiping Min

**Affiliations:** ^1^Institute of Immunotherapy and College of Basic Medicine of Nanchang University, and Jiangxi Academy of Medical Sciences, Nanchang, China; ^2^Medical Science Laboratory, Children's Hospital, Maternal and Child Health Hospital of Guangxi Zhuang Autonomous Region, Nanning, China; ^3^Jiangxi Provincial Key Laboratory of Immunotherapy, Nanchang, China; ^4^Department of Endocrinology and Metabolism, Peking University People's Hospital, Beijing, China; ^5^Department of Oncology, The First Affiliated Hospital of Nanchang University, Nanchang, China; ^6^Medical Laboratory, Jiangxi Provincial People's Hospital, Nanchang, China; ^7^Department of Surgery, Pathology and Oncology, University of Western Ontario, London, ON, Canada

**Keywords:** GNR (gold nanorods), LLC (lewis lung cancer), PTT (photothermal therapy), LSPR (localized surface plasmon resonance), IDO (Indoleamine 2, 3-dioxygenase)

## Abstract

**Background:** Photothermal therapy (PTT) has been demonstrated to be a promising cancer treatment approach because it can be modulated to induce apoptosis instead of necrosis *via* adjusting irradiation conditions. Recently, an abscopal anti-tumor immunity has been highlighted, in which PTT on the primary tumor also induced repression of distant tumors. In PTT cancer treatments, the mechanism and the role of immune checkpoints to enhance anti-tumor immunity needs to be investigated.

**Methods:** We prepared a multi-functional gold nanorod reagent, GMPF-siIDO, that is composed of gold nanorods (GNRs) that act as the nano-platform and photothermal sensitizer; folic acid (FA) as the tumor-targeting moiety; and IDO-specific RNA (siIDO) as an immune-stimulator functionality for inducing anti-tumor immunity. For this study, we adjusted the irradiation condition of PTT to induce apoptosis and to silence the immune checkpoint indoleamine 2,3 dioxygeonase (IDO), simultaneously.

**Results:** Our studies provide evidence that photothermal effects kill tumor cells mainly *via* inducing apoptosis, which can significantly improve antitumor immunity when IDO was down-regulated in TME through significant increases of localized CD8^+^ and CD4^+^ lymphocytes in tumor tissue, the downregulation of CD8^+^ and CD4^+^ lymphocyte apoptosis, and the upregulation of antitumor cytokines, TNF-α and IFN-γ.

**Conclusion:** In this study, we, for the first time, validated the role of IDO as a negative regulator for both PTT-induced tumor cell apoptosis and anti-tumor immunity; IDO is a critical immune checkpoint that impedes PTT while combination of gene knockdown of IDO in TME enhances anti-tumor efficacy of PTT.

## Introduction

Cancer, especially solid tumors, has become a major global health threat in the twenty-first century. Lung cancer ranks first in incidence and mortality rates in cancer patients (1). Many studies have shown that various malignant tumors highly express Indoleamine 2,3-dioxygenase-1 (IDO) (2–4). IDO is a critical endogenous immune suppressive factor, which exhausts tryptophan levels of T cells to produce kynurenic acid. This results in suppression of the activation of T cells in a cancer milieu (5). IDO is also known to facilitate the generation of regulatory T cells, which further attenuates anti-tumor immune action (6, 7). IDO, as an immune checkpoint, is a major hurdle for anti-tumor immunity and immunotherapy for cancers. Our previous studies have shown that gene silencing of IDO, through RNA interference, can stimulate anti-tumor immunity and inhibit tumor angiogenesis, thereby resulting in the killing of tumor cells and suppressing tumor invasion, metastasis, and growth (8, 9). These results make IDO a promising targeting molecular cancer immunotherapy.

In recent years, near-infrared (NIR) light-mediated photothermal therapy (PTT) has become attractive for cancer therapy because of low invasiveness and high specificity of the treatment (10–12). PTT can induce tumor cell apoptosis or necrosis, thus inhibiting tumor growth through localized hyperthermia (13, 14). Among various light absorbers in photothermal therapy, gold nanorod (GNR) is capable of variable longitudinal surface plasmon resonance (LSPR) and shows high efficiency in converting the photothermal effect of the near-infrared spectrum. It is widely used in PPT-based cancer treatments under study *in vivo* (12, 14). More recently, combination treatment of PTT with other therapeutic strategies such as chemotherapy has demonstrated enhanced clinical efficacy by upregulating the systemic immune anti-tumor system (15). However, PTT-treatment also elevated the expression of IDO, which suppresses tumor cell apoptosis as well as impairs anti-tumor immunity, resulting in impediment of PTT therapy efficacy (16). Therefore, a new strategy of PTT which can simultaneously knockdown negative immune regulator IDO to suppress tumor-derived immune suppression while increase tumor cell apoptosis would be beneficial.

In this study, we attempted to deliver IDO siRNA (siIDO) to lung tumor cells for immuno-photothermal therapy using a novel gold nanorod reagent, GMPF-siIDO. Our results indicate that GMPF-siIDO could effectively deliver siRNA for the IDO gene into LLC tumor cells with high specificity *in vitro* and *in vivo*. We demonstrated that silencing IDO not only increased LLC cell apoptosis under PTT, but also enhanced the anti-tumor immune response. Treatment with GMPF-siIDO synergized PPT in suppressing tumor growth *in vivo* with high efficacy, minimal invasiveness, and minimal normal tissue side-effects, providing evidence for a novel combined targeted strategy as a fundamental principle for personalized medicine in the treatment of lung cancer.

## Materials and Methods

### Animal Usage

Female C57BL/6 mice, 6–8 weeks of age and weighing 18–20 g, were purchased from Changsha Animal Laboratory (Changsha, China). All mice were housed in a specific pathogen free (SPF) grade animal center. The use of the mice complied with the Regulations for the Administration of Affairs Concerning Experimental Animals of China. All animal experiments received the ethical approval of Animal Care and Use Committee of Nanchang University, China.

### Synthesis of GNRs

Soluble gold nanorods were synthesized using a previously published seed-mediated growth protocol (17). Briefly, a seed solution was first prepared by mixing 1 mL of cetyltrimethylammonium bromide (CTAB) solution (0.2 M) with 1 mL of HAuCl_4_ (0.5 mM), followed by the addition of 0.12 mL of ice-cold 0.01 M NaBH_4_ until the resulting seed solution turned in to brownish yellow color. Secondly, 2.5 mL of AgNO_3_ (4 mM) was added to the mixture containing 50 mL of HAuCl_4_ (1 mM) and 50 mL of CTAB (0.2 M). Then, 670 mL of ascorbic acid (0.079 M) was added, making the solution colorless. Finally, 120 mL of the seed solution was added to the growth solution for 24 h, followed by a centrifuge (14,000 g for 15 min) to remove the excess of CTAB. The nanorod was characterized by UV-vis-NIR spectrometer and transmission electron microscope (TEM).

### Synthesis of GNR-MUA-PEI (GMP) and GNR-MUA-PEI-FA (GMPF)

Synthesis of MUA-PEI and MUA-PEI-FA is in accordance with the protocol as we described previously (18). In brief, the synthesis steps included (1) carboxyl groups of MUA reacting with amino groups of polyethyleneimine (PEI), by ethyl (dimethylaminopropyl) carbodiimide (EDC)-mediated amidation of N-hydroxysuccinimide (NHS), in the presence of HCCl_3_, (2) carboxyl groups of folic acid (FA), reacting with the remaining free amino groups of PEI by the similar steps of EDC-mediated amidation with NHS, but in presence of DMSO instead of HCCl_3_. The MUA-PEI moiety replaced CTAB on GNRs to produce the GNR-MUA-PEI species, and 1:1 ratio of MUA-PEI to MUA-PEI-FA replaced CTAB on GNRs to form the GNRs-MUA-PEI-FA species of nanocarriers. The products were characterized by Hydrogen Nuclear Magnetic Resonance (HNMR), UV-vis-NIR spectrometer and TEM.

### siRNA Synthesis

The siRNAs specific for IDO or GAPDH mRNA were designed and synthesized based on previously published methods (19, 20). siRNAs were synthesized commercially (Sigma, St. Louis, MO). Luciferase GL2 siRNA) was synthesized (Sigma) and used as a silencing negative control.

### Gel Shift Assay

The GMPF-siRNA nano-complexes with different ratios of GMPF to siRNA were prepared by mixing 0.6 μg IDO siRNA and varying amounts of GMPF. The weight ratios of siRNA to GMPF used were 1:0, 1:0.5, 1:1, 1:1.5, 1:2, 1:2.5, 1:3, 1:3.5, 1:4, 1:4.5, and 1:5. The components were mixed and incubated for 30 min, followed by electrophoresis at 80 V in 1.5% agarose gels in TAE (Tris-acetate-EDTA) buffer, supplemented with ethidium bromide. The gels were visualized under UV illumination and imaged using an Olympus C8080 camera system.

### Release of siRNA From GMPF

An established gel-shift method was applied to evaluate the release of siRNA from GMPF-siRNA (21). All the above prepared GMPF samples, with various ratios of GMPF-siRNA, were incubated with 4 μL of 2% sodium dodecyl sulfate (SDS) for 10 min. The siRNA release of from GMPF was determined by band-shifts in 1.5% agarose gel after electrophoresis.

### GMPF-siRNA Stability in Serum

GMPF-siIDO aliquots were incubated in 50% FBS at 37°C for 0, 4, 8, 24, 48, and 72 h, then immediately mixed with 2% SDS-containing gel loading buffer. Samples underwent 1.5% agarose gel electrophoresis to assess disconjugation of the nano-complex moieties as indicated by band-shifts.

### Photothermal Conversion Measurement

The photothermal efficiency was measured on a customized in-house laser equipment. 24-well microplate with 2 mL of the nano-carrier samples were prepared. A fiber-coupled continuous semiconductor laser (808 nm) with a power density of 2 W/cm^2^ was applied. The temperatures of the solutions were measured. Each sample was irradiated for 10 min and the temperature was recorded at 0, 1, 2, 3, 4, 5, and 10 min.

### Cell Culture

The Lewis lung cancer (LLC) cells were obtained from the American Type Culture Collection (ATCC). The cells were cultured in DMEM medium (Invitrogen Life Technologies, Carlsbad, CA, USA) with 10% FBS and standard amounts of L-glutamine, penicillin, and streptomycin at 37°C in 5% CO_2_, using a routine method (22).

### Cell Uptake of GMPF-siRNA

The uptake efficiency of siRNA delivered by the GMPF construct was determined by flow cytometry and fluorescence microscopy as described previously (18). Briefly, the same amounts (1 μg) of Cy3-siGAPDH siRNA were mixed with various concentrations of GMPF at 10, 20, 40, and 80 μg/mL. These GMPF-siRNA mixtures were added to LLC cells (5 × 10^4^ cells) in 24-well microplates. After 24 h, the internalization of siRNA in the cells was confirmed by fluorescent microscopy. The cells were trypsinized, harvested, washed (PBS), and analyzed by flow cytometry (BD FACS Calibur, BD Biosciences, Mountain View, CA).

### Gene Silencing and Quantitative Real-Time Quantitative PCR (qRT-PCR)

LLC cells were seeded in a 12-well plate (10^5^ cells/well) and transfected with a final concentration of 40 μg/mL of GMPF-siRNA [wt(FA-GNR):wt(siRNA) = 30:1, wt(siRNA) = 0.6 μg] for 24 h. The total RNA was prepared and used to synthesize cDNA using reverse transcriptase (MMLV-RT, Invitrogen Life Technologies). The following primer sets were used for q-PCR amplifications: β-Actin, 5'-AGGGAAATCGTG CGTGACAT-3' (sense) and 5'-AACCGCTCGTTGCCA ATAGT-3' (antisense); IDO, 5'-GTACATCACCATGGCGTATG-3' (sense) and 5'-CGAGGAAGAAGCCCTTGT C-3' (antisense). The qRT-PCR was conducted using SYBR green PCR reagents (Invitrogen Life Technologies). The reactions of qRT-PCR were amplified, in accordance to the manufacturer's protocol, in Stratagene Mx3000P QPCR System (Agilent Technologies, Santa Clara, CA, USA). The difference in gene expressions in treatment groups were calculated using the ΔCt method.

### Western Blot Analysis

Cells (1 × 10^5^/well) were seeded into a 12-well plate and were transfected with a final concentration of 40 μg/mL of GMPF-siRNA [wt(FA-GNR):wt(siRNA) = 30:1, wt(siRNA) = 0.6 μg] for 48 h. Cells were harvested and lysed after transfection of GMPF-siRNA. The cell lysates were separated on a 10% SDS-PAGE, and then transferred to nitrocellulose membrane. The membranes were probed with a mouse anti-human IDO mAb (Santa Cruz Biotechnology, Santa Cruz, CA, USA) and anti-β-actin mAb (Santa Cruz Biotechnology) according to the manufacturer's instructions. The membranes were finally visualized by an ECL assay kit (Pierce, Rockford, lL, USA).

### Bio-Distribution of GMPF-siRNA *in vivo*

Mice bearing established tumors were hydro-dynamically injected with 200 μL GMP-Cy3-siGAPDH (containing 200 μg GMP and 30 μg Cy3-siGAPDH) or GMPF-Cy3-siGAPDH (containing 200 μg GMPF and 30 μg Cy3-siGAPDH). 24 h post-administration, all mice were sacrificed and the tumors, heart, liver, spleen, lung, and kidney were isolated and frozen using an optimal cutting temperature (OCT) compound (Triangle Biomedical Sciences, USA); it was then sliced into 7 μm horizontal sections. Images were captured using a fluorescence microscope (Olympus, Model BX 51, Japan).

### Treatment of Lung Cancer Using Photothermal-Immunotherapy

The cancer-bearing mice were generated by inoculating 5 × 10^5^ LLC cell suspensions at subcutaneous tissues of the mice's back. When the diameter of tumor was ~3 mm, the mice were randomized into five groups. The mice were hydro-dynamically injected with 400 μL PBS (control), GMPF-siGL2 or GMPF-siIDO (containing 400 μg GMPF and 50 μg siRNA) at day 4, 9, 14, 19. 24 h after each injection, the treated mice were irradiated by the 808 nm near-infrared laser irradiation (1 W/cm^2^, 5 min). The length (L) and width (W) of tumor diameters were measured every other day using a digital caliper. The tumor volumes were calculated using a formula: V =1/2 (L × W^2^). At day 22 after tumor inoculation, the mice were sacrificed and tumors, blood, and spleens were collected. The tumor weights were measured, and images were taken with a camera.

### Immunohistochemistry

The tumor tissues were collected, fixed in 10% formalin and sectioned into 5-μm slices. After blocking endogenous peroxidase using in 3% H2O2-methanol,block the slides were incubated with 5% normal goat serum 60 min and stained with rat anti-IDO, anti-CD8, or anti-CD4 polyclonal antibody (1:50, Santa Cruz Biotechnology) overnight at 4°C. The slides were then incubated with biotinylation secondary antibody at 37°C for 60 min, following an incubation with horseradish peroxidase (HRP)-labeled streptavidin at 37°C. After development, using diaminobenzene (DAB), the sample slides were observed and photographed under microscope. The integrated optical density (IOD) of IDO were measured in immunostained sections, following the instructions of the Image Pro Plus 6.0 software (Media CY Company, USA).

### TUNEL Assay

The tumor tissues were frozen in liquid nitrogen and then sectioned into 5 mm segments. The *in-situ* cell death was detected by a TUNEL assay kit (7 Sea Pharmtech, Shanghai, China), following the manufacturer's instructions. The prepared specimens were examined using a light microscope.

### ELISA Assay

Triplicate serum samples (100 μL/well) were collected from experimental mice and placed in mAb (against TNF-α or IFN-γ)-precoated microtiter plates overnight. The plates were blocked with 5% BSA for 1 h at 37°C, then with Biotin-labeled secondary mAb (against TNF-α or IFN-γ, respectively) for 1 h at 37°C, followed by four washes with PBS buffer. After incubation with Strepavidin-HRP 1 h at 37°C, the samples were washed and treated with ELISA solutions (Liankebio, Hangzhou, China). The plate signals were read at 450 nm using an ELISA Reader (SpectraMax, MD, USA). Concentrations of TNF-α and IFN-γ in samples were calculated after calibrating the standard curves that were plotted as absorbance vs. logarithm of the analyte concentration.

### Flow Cytometry

Lymphocytes isolated from collected spleens were incubated with anti-CD4 and anti-CD8 antibodies at 4°C for 30 min. These anti-CD4 and anti-CD8 stained T cells were further stained with Annexin V (eBioscience, USA) at 4°C for 15 min. Cells were sorted by flow cytometry using FACS Calibur II (BD Biosciences).

### Statistics

Data is presented as mean ± SD (Standard deviation). Student's *t*-test (2-tailed) was used to determine differences between two means. To compare multiple groups, a one-way ANOVA test was applied. A difference with a *p* < 0.05 was considered significant.

## Results

### Photothermal Effects of Multi-Functionalized Gold Nanorod-MUA-PEI-Folate (GMPF)

**Figure 1:**
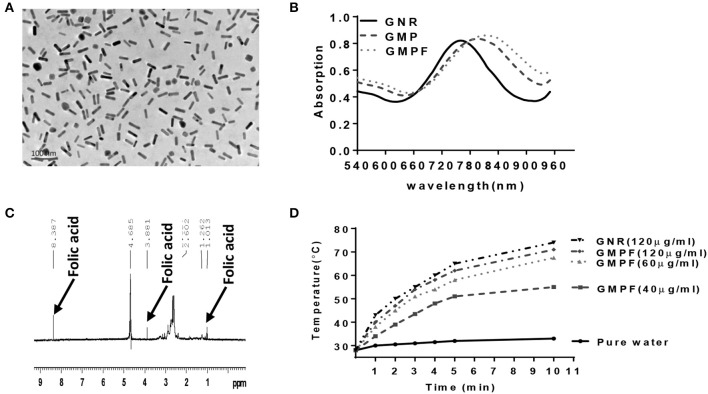
Characterization of photo-thermal effects of a multi-functionalized gold nanorod GNR-MUA-PEI-FA, GMPF. **(A)** Transmission Electron Microscopy (TEM) image of modified GNRs, GMPF. **(B)** UV-Vis absorption spectra of GNR modified by CTAB (GNR), or MUA-PEI (GMP), or MUA-PEI-FA (GMPF). **(C)** The chemical signature of folic acid on the surface of GNR determined by NMR after GNR conjugation with folic acid. **(D)** Temperature increase induced by photothermal effects of GNR or GMPF *in vitro*. 2 mL of GNR (120 μg/mL) or GMPF (40, 60, 120 μg/mL), or deionized water was irradiated at 2 W/cm^2^ with the laser wavelength at 808 nm for 10 min. The temperatures were measured and plotted at indicated time points.

CTAB-stabilized GNRs were synthesized as previously described using a seed-mediated growth method. Transmission electron microscopy (TEM) was used to analyze the morphology of GNRs. The GNRs were 10 × 40 nm (width x length) (Figure 1A). As shown in Figure 1B, the original GNRs exhibited two SPR peaks: a weak transverse SPR (TSPR) peak at ~525 nm and a strong peak of longitudinal SPR (LSPR) at ~770 nm. A peak with slight red-shifting to 785 nm was observed from the LSPR curve for GMP after the MUA-PEI modification process. This was followed with a further red-shift to 790 nm of the LSPR curve of GMPF, which suggests that GMP and GMPF were successfully synthesized. It has been reported that the shift of LSPR band peak from the GNRs result from the local refractive index around the GNRs, which is sensitive to the changes on the surface of the GNRs (23). Next, we detected GMPF using ^1^H NMR spectra. As shown in Figure 1C, peaks at δ1.013, δ3.881, and δ8.387 suggest that folic acid (FA) was successfully conjugated on the surface of GMP. FA was used as a tumor-targeting moiety on GMPF, as most tumor cells such as those found in lung cancers, breast cancers, and melanomas highly express FA receptors (24–27).

Research has shown that gold nanorods are promising candidates for photothermal therapy under NIR irradiation (12, 14). Therefore, we determined the photothermal effects of the GMPF under the 808 nm laser irradiation. An increase of temperature to 74 and 71°C from room temperature, after 10 min of NIR laser irradiation of GNRs (120 μg/mL) and GMPF (120 μg/mL), respectively, was recorded (Figure 1D). The temperature increase gradients were associated with the GMPF concentrations. In contrast, the temperature in pure water after NIR irradiation only showed a marginal increase to 33°C. These results indicate that the GMPF possess excellent photothermal properties. We next used 40 μg/mL of GMPF and irradiation condition of 2 W/cm^2^ for 5 min to treat the tumor cell *in vitro*. This treatment elicited temperature increases up to 50°C, which is optimal for apoptotic and necrotic killing of tumor cells (28, 29).

### Gene Silencing of IDO Using GMPF-siIDO

**Figure 2:**
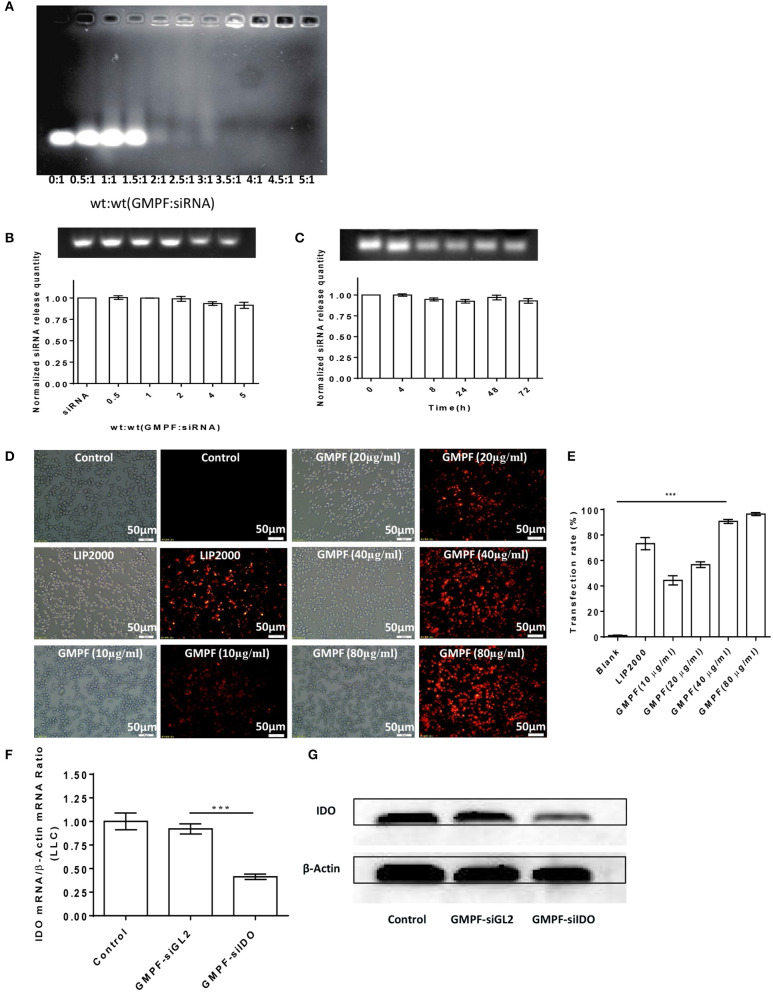
Bio-characterization of GMPF-siIDO. **(A)** Gel shift assays were used to determine the siRNA-loading capacity of GMPF. Equal volumes containing 0.6 μg of siRNA and the desired amount of GMPF were incubated at the indicated weight ratios for 30 min, run on agarose gels, and visualized with ethidium bromide. **(B)** Determination of siRNA release from GMPF-siIDO using SDS analysis. Various ratios of GMPF to siRNA [wt(siRNA) = 0.6 μg] were prepared and all the samples were treated with 4 μL of 2% SDS and incubated for 10 min at room temperature. Release of siRNA from GMPF-siIDO was determined by electrophoresis (upper panel). The relative quantity of released siRNA was, respectively, calculated by released siRNA/total siRNA (lower panel). **(C)** Serum stability of GMPF-siIDO. GMPF-siIDO [wt(GMPF): wt(siRNA) = 4:1, wt(siRNA) = 0.6 μg] was incubated in 50% FBS at 37°C for 0, 4, 8, 24, 48, and 72 h, and then incubated with 2% SDS. The SDS-treated samples were subjected to gel electrophoresis. The quantity of stable siRNA was calculated by serum-incubated siRNA /non-serum incubated siRNA. **(D,E)** Targeted siRNA transfection efficiency by GMPF. Cy3-labeled siRNA (1 μg) were transfected into LLC by PBS as control, lipofectamine 2000 (LIP2000), GMPF with various concentrations 10, 20, 40, and 80 μg/ml. After 24 h incubation, the intracellular fluorescence intensity was observed under fluorescence microscope **(D)** (scale bar = 50 μm) and the transfection rates were measured using flow cytometry **(E)**. **(F,G)** *in vitro* gene silencing using GMPF-siIDO. LLC cells were incubated with a final concentration of 40 μg/mL of GMPF-siIDO or GMPF-siGL2 (non-specific control siRNA) [wt(FA-GNR): wt(siRNA) = 30:1, wt(siRNA) = 0.6 μg] or PBS as the null treatment control for 24 h. Transcript levels of IDO were quantified by qRT-PCR. Error bars represent the standard deviation of three experiments (**P < 0.01) **(F)**, or 48 h after transfection, the protein levels of IDO and β-actin was determined by western blot **(G)**. Error bars represent the standard deviation of three experiments (***P < 0.001).

We next evaluated the ability of GMPF carrying IDO siRNA based on the fact that the free siRNA migrates faster, while GMPF-bound siRNA is nearly immobile due to electrostatic interactions (30), and the gel shift visually represents the capacity of GMPF binding to siIDO. The results showed that when the ration of GMPF to siIDO reached 3.5:1 (w/w), unbound siIDO was not observed to be above this ratio (Figure 2A). These data suggested that the effective ratio required to saturate GMPF with siIDO is 3.5:1.

To test whether bound siRNA can be released from GMPF, the release profile of siRNA from GMPF-siRNA complexes was determined by a SDS replacement assay (21). Figure 2B shows that siRNA is effectively released from GMPF, with a more than 91.5% release.

Previous reports suggest that free siRNA is easily degraded in serum and stable for only 6 h (31, 32). We tested whether GMPF protected bound siRNA from degradation by enzymes in serum. The stability of GMPF-bound siRNA was evaluated after incubating GMPF-siRNA with 50% FBS at various time-points (i.e., 0, 4, 8, 24, 48, 72 h), and was then examined by SDS replacement. As shown in Figure 2C, the GMPF-siRNA samples had visible bands, suggesting that about 93.0% siRNA were not degraded even after 72 h of incubation in serum. This result indicates that the GMPF construct can protect siRNA against enzymatic degradation *in sera* longer than previously reported.

Next, we assessed the cellular uptake of the GMPF-siRNA by LLC cells. The red fluorescence of Cy3 was observed in LLC cells incubated with the GMPF-Cy3-siGAPDH (Figure 2D). In contrast, the cells treated with Cy3-siGAPDH in PBS show no red fluorescent signals. We observed that an increase of red fluorescent signals correlate to the increase of the GMPF concentrations from 10 to 80 μg/mL (Figure 2D). Compared to the control cells, 92.3% of cells transfected with GMPF-cy3-siGAPDH (40 μg/mL) were red fluorescence positive, which is significantly higher than the cells transfected with the conventional transfection reagent Lipofectamine 2000 (75.9%; Figure 2E). This suggests that the GMPF is a tumor-targeted and effective nano-carrier for transfecting siRNA into LLC cancer cells.

Finally, we determined the gene silencing efficiency of GMPF-siIDO in tumor cells. The transfection with GMPF-siIDO significantly knocked down IDO expression in LLC cells by 60% after (Figure 2F), as compared to control transfection with GMPF-siGL2. The knocking down of IDO at the protein level was also obvious in LLC cells (Figure 2G).

### Gene Silencing of IDO Using GMPF-siIDO Enhances Photothermal Effects of the Multifunctional Gold Nanorods

**Figure 3:**
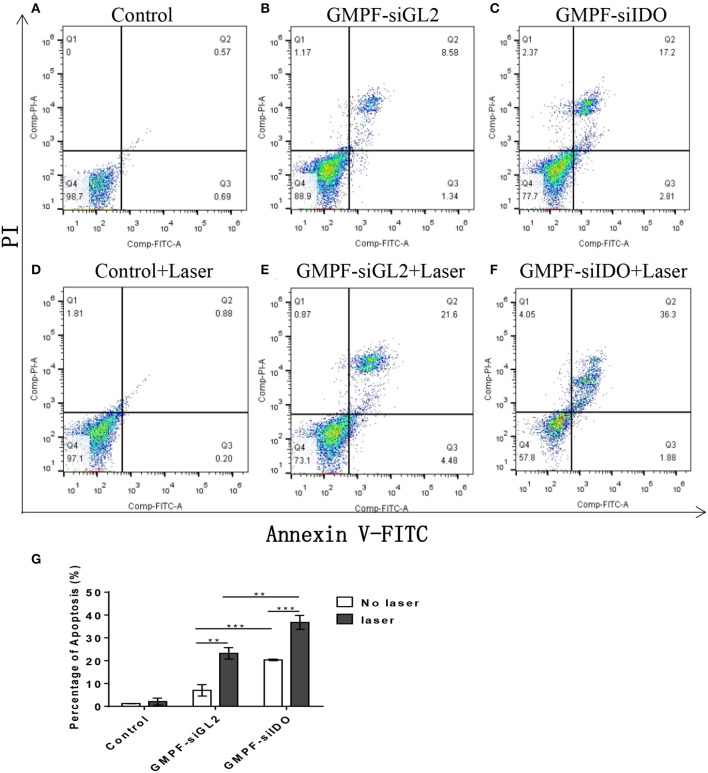
Assessment of tumor cell apoptosis by photo-thermal effects of GMPF-siIDO *in vitro*. LLC cells were incubated with 40 μg/mL of GMPF-siGL2 or GMPF-siIDO overnight, and samples were then irradiated at 2 W/cm^2^ for 5 min. The apoptotic cell distribution of all was determined at 24 h using the Annexin V-FITC/PI Apoptosis Detection Kit and analyzed by flow cytometry **(A–F)**, and the percentages of apoptosis are **(G)**. Error bars represent the standard divination of three experiments (**p < 0.01; ***p < 0.001).

PTT can induce apoptosis instead of necrosis *via* adjusting the irradiation conditions to kill tumor cells (29). Our previous studies have demonstrated that gene silencing of IDO2 can induce B16-BL6 tumor cell apoptosis (18, 33). In this study, we evaluated whether GMPF-siIDO could enhance tumor apoptosis in the combined condition of silencing IDO along with the photothermal effect induced by gold nanorods. As shown in Figure 3, while treatment with control siRNA silencing (Figure 3B) alone did not alter the ratio of apoptosis as compared to the untreated control tumor cells (Figure 3A), however, knockdown of IDO significantly increased apoptosis of tumor cells (Figure 3C). On the other hand, laser irradiation alone, without GMPF nanoparticles, did not induce apoptosis (Figure 3D), and significant apoptosis was displayed in the tumor cells in the presence of GMPF after laser irradiation (Figure 3E). Most significantly, the combination of GMPF-siIDO and laser irradiation treatments achieved the highest ratio of apoptosis in tumor cells (Figure 3F). The statistical analysis showed that combination treatment induced significantly more apoptotic cells (36.8 ± 3.06%) than individual treatment witheither silencing of IDO (20.4 ± 0.35%) or laser irradiation (23.2 ± 2.52%) (Figure 3G).

### *In vivo* Tumor-Targeted Photothermal-Immunotherapy for Lung Cancer Using GMPF-siIDO

**Figure 4:**
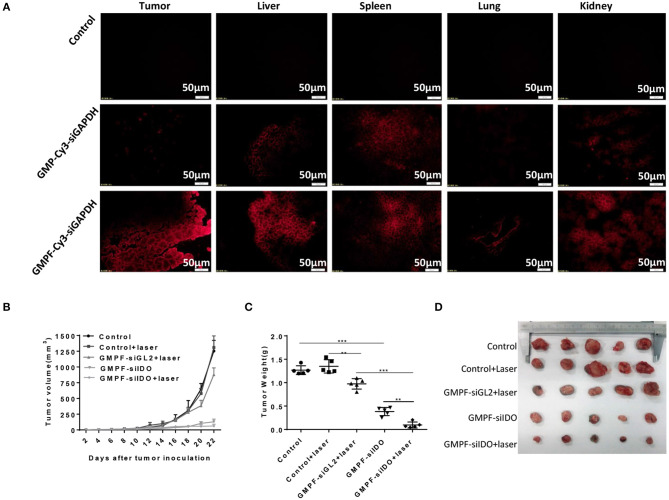
Tumor-targeted photothermal-immunotherapy for lung cancer using multi-functionalized gold nanorods, GMPF-siIDO. **(A)** *in vivo* targeted delivery of GMFP-siGAPDH. LLC tumor bearing mice were intravenously injected with PBS (control), Cy3-labeled GMP-siGAPDH or GMFP-siGAPDH. 24 h post administration, mice were sacrificed and the tumors, livers, spleens, lungs, and kidneys were isolated for frozen tissue sectioning. The fluorescent intensity of tissue sections was visualized using fluorescence microscopy. The data represents one of three independent experiments (Scale bar = 50 μm). **(B–D)** Anti-tumor effects of GMPF-siIDO. LLC lung cancer bearing mice were intravenously injected with PBS (control), GMPF-siGL2 or GMPF-siIDO on day 4, 9, 14, and 19 after tumor inoculation. 24 h after each treatment, the mice were irradiated by the 808 nm near-infrared laser at 1 W/cm^2^ for 5 min. The tumor sizes were measured for 22 days after tumor inoculation **(B)**. At the end point of observation, the mice were sacrificed, and tumors collected. The tumor weights were measured **(C)** and the images of tumors are displayed in **(D)**. Error bars represent the standard deviation of three experiments (**P < 0.01; ***P < 0.001).

To assess *in vivo* tumor-targeted siRNA delivery by GMPF, we injected Cy3-labeled GMPF-siRNA into LLC lung cancer-bearing mice for the *in vivo* observation of GMPF distribution. The results showed that red fluorescence was displayed in liver, lungs, kidneys, and spleen, suggesting that the siRNA carried by both targeted and non-targeted nanoparticles were distributed to the blood-rich organs. However, for the delivery of tumor tissues, only GMPF-siRNA was markedly accumulated in tumors compared to control or by non-targeted GMP-siRNA (Figure 4A).

To evaluate the anti-neoplastic therapeutic efficacy of immunostimulatory multi-functional gold nanorods, we treated the LLC cancer-bearing mice with GMPF-siIDO. The tumor grew aggressively in the mice sham-treated with PBS or PBS with irradiation (Figure 4B). The treatment with non-specific siRNA-conjugated gold nanorods (GMPF-siGL2) only achieved minor suppression of tumor growth, however, the treatment with GMPF-siIDO significantly delayed the tumor growth by day 22 post-inoculation of tumor cells (Figure 4B). The anti-tumor effect was enhanced when the lung cancer bearing mice received a combination treatment with GMPF-siIDO and laser irradiation, which further suppressed tumor growth (Figure 4B). The therapeutic efficacy of combination treatment of GMPF-siIDO and laser irradiation was further confirmed by the tumor weight (Figure 4C) and size (Figure 4D), which were remarkably smaller (0.09 ± 0.064 g) than that in the mice that received treatment with laser irradiation (0.97 ± 0.11 g) or gene silencing of IDO (0.38 ± 0.08 g).

### Tumor-Targeted Gene Silencing of IDO Enhanced Apoptosis of Tumor Cells After Photothermal-Immunotherapy *in vivo*

**Figure 5:**
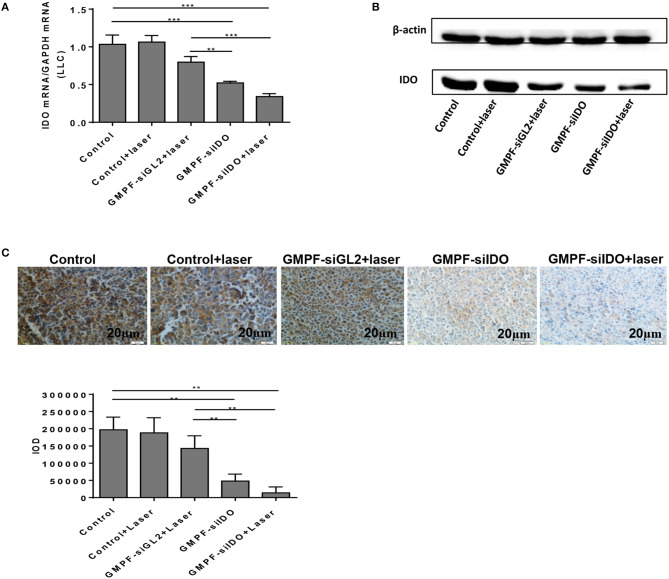
Tumor-targeted gene silencing of IDO using GMPF-siIDO *in vivo*. Tumor-targeted gene silencing. Lung cancer-bearing mice were treated with PBS (control), GMPF-siGL2, or GMPF-siIDO, with or without laser irradiation. At the endpoint of the experiment, the mice were sacrificed, and tumor tissues were collected. The IDO expression was detected by RT-qPCR **(A)**, western blot **(B)**, and immunohistochemistry assays **(C)**. Error bars represent the standard deviation of three experiments. Error bars represent the standard deviation of three experiments (**P < 0.01; ***P < 0.001).

To confirm the siIDO carried by GMPF-siIDO was functional *in vivo*, we determined the gene knockdown of IDO in the above treated mice. The IDO expression in tumors significantly declined after the treatment of GMPF-siIDO compared to the control or GMPF-siGL2 (Figure 5A). The repression of IDO expression was also confirmed at the protein level (Figure 5B) and immunochemistry (Figure 5C). These results highlight that GMPF-siIDO is effective in knocking down IDO gene *in vivo* as well.

**Figure 6:**
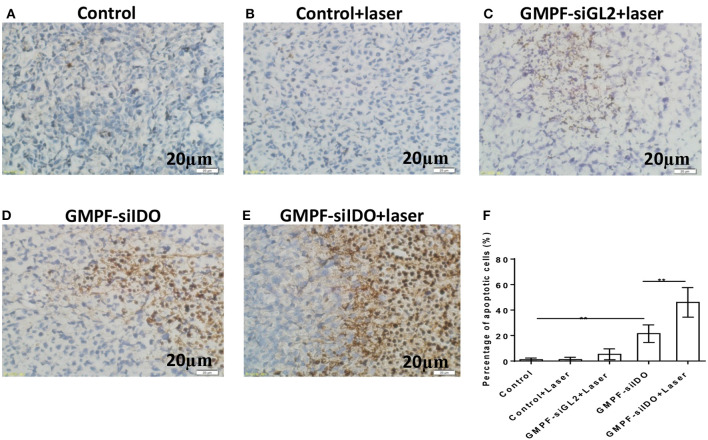
Enhanced apoptosis of tumor cells by photothermal-immunotherapy. LLC lung cancer-bearing mice were treated with PBS (control), GMPF-siGL2, or GMPF-siIDO, with or without laser irradiation. Tumor tissues were collected, and apoptosis was detected by TUNEL assays **(A–E)** and percentages of apoptotic cells were calculated **(G)**. Error bars represent the standard deviation of three experiments. Error bars represent the standard deviation of three experiments (**P < 0.01).

The recently developed GMPF-siIDO possesses multiple advantages: tumor-specific targeting *via* the FA moiety, siRNA-based IDO gene knockdown, and GNR-induced photothermal tumor-killing effects. As gene silencing of IDO promotes tumor cell apoptosis, photothermal therapy (PTT) with GNRs also directly kill the tumor cells through inducing apoptosis. Therefore, we tested whether the GMPF-siIDO may synergize the gene silencing of IDO and photothermal effects in induction of apoptosis of tumor cells *in vivo*. As shown in Figure 6, the apoptosis rates in the untreated tumor (Figure 6A) and control laser irradiation (Figure 6B) were low. In contrast, combination treatment of GMPF-siIDO and laser irradiation induced the highest level of tumor cell apoptosis (49.11 ± 2.26%, Figure 6E), while laser irradiation (8.40 ± 1.52%, Figure 6C) or GMPF-siIDO treatment (22.06 ± 1.84%, Figure 6D) alone showed lower efficiency in inducing apoptosis off tumor cells. These data verified that knockdown of the checkpoint regulatory molecule IDO can synergize *in vivo* apoptosis of the tumor cell.

### Increase of Tumor Infiltrating T Cells and Upregulation of Inflammatory Cytokines After GMPF-siIDO Photothermal-Immunotherapy Treatment

**Figure 7:**
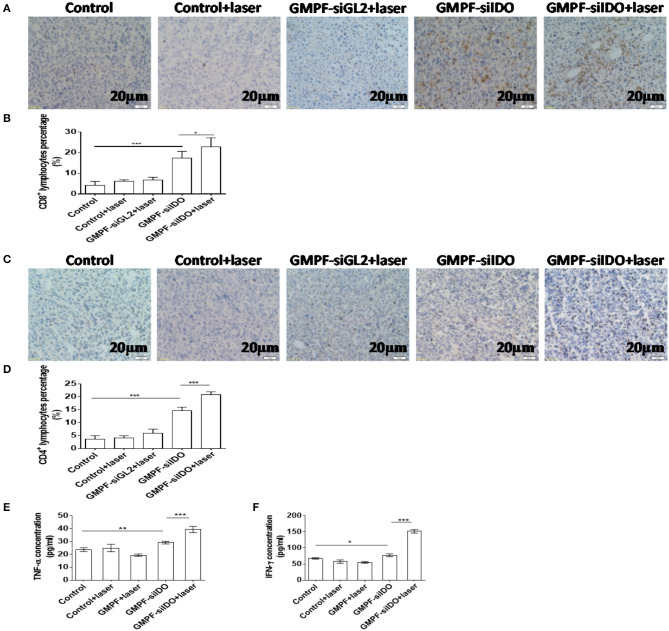
Increased tumor-infiltrating T cells and up-regulated inflammatory cytokines after photothermal-immunotherapy. **(A–D)** Increased TILs in tumor tissue. Lung cancer-bearing mice were treated with PBS (control), GMPF-siGL2 or GMPF-siIDO, with or without laser irradiation. Tumor tissues were collected and subjected to immunohistochemistry assays and CD8^+^ **(A,B)** and CD4^+^ **(C,D)** lymphocytes in tumor tissue were counted. **(E,F)** Upregulated inflammatory cytokines. At the endpoint of experiment, the mice were sacrificed, and total blood was also collected. The TNF-α **(B)** and IFN-γ **(C)** in blood were analyzed by ELISA assay. Error bars represent the standard deviation of three experiments (*P < 0.05; **P < 0.01; ***P < 0.001).

IDO is a primary endogenous immunosuppressive factor. It facilitates the tumor cell escape from immune privilege *via* exhausting the tryptophan in T cells used to produce kynurenic acid, thus inhibiting activation of T cells and inducing regulatory T cells. Therefore, we posited that the multi-functional gold nanorod-based reagent GMPF-siIDO could specifically silence IDO expression in tumor tissues leading to an increase of the number of tumor-infiltrating T cells and the secretion of anti-tumor inflammatory cytokines. We analyzed the infiltrated T cells (TILs) in the tumor tissues. As shown in Figures 7A–D, the CD8^+^ and CD4^+^ TILs were markedly increased after the combination treatment with GMPF-siIDO and laser irradiation. Untreated mice showed few CD8+ TILs inside the tumor (4.28 ± 1.83%). The TILs, after combination treatment, were notably increased (22.91 ± 4.28%), which were significantly higher than that in the tumors from the mice treated with laser irradiation alone (6.73 ± 1.32%) or with IDO silencing alone (17.30 ± 3.35%) (Figure 7B). Similarly, the CD4^+^ TILs in the tumor mice after combination treatment with GMPF-siIDO and laser irradiation (20.85 ± 1.05%) were significantly higher than that in the tumors from the un-treated mice (3.72 ± 1.25%), mice treated with laser irradiation alone (5.87 ± 1.62%) or with IDO silencing alone (14.52 ± 1.46%) (Figure 7D). These results indicate that gene silencing IDO was capable of synergizing PTT-induced T cell infiltration, thus, enhancing anti-tumor immunity in the combination treatment of GMPF-siIDO in lung cancer.

Additionally, anti-tumor inflammatory cytokines TNF-α and IFN-γ in serum were increased by 2.1-fold (39.38% ± 2.41 pg/mL vs. 19.47 ± 0.92 pg/mL) and 2.8-fold (151.9 ± 5.66 pg/mL vs. 55.34% ± 3.01 pg/mL) after the treatment with GMPF-siIDO and laser irradiation, respectively, compared to the treatment of GMPF and laser irradiation without IDO silencing (Figures 7E,F).

### Suppression of T-Cell Apoptosis by Photothermal-Immunotherapy Using GMPF-siIDO

**Figure 8:**
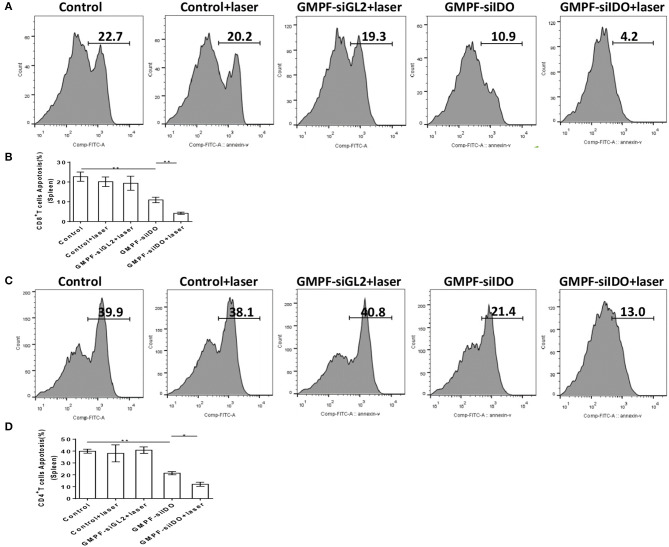
Suppression of T cell apoptosis by photothermal-immunotherapy. Lung cancer-bearing mice were treated with PBS (control), GMPF-siGL2, or GMPF-siIDO, with or without laser irradiation. T cells were isolated and apoptosis of CD4^+^ **(A)** and CD8^+^ **(C)** T cells in spleens were analyzed by flow cytometry after Annexin V staining. The correspondent apoptotic percentages of CD8^+^ T cells **(B)** and CD4^+^ T cells **(D)** were calculated and results represent one of three experiments. Error bars represent the standard deviation of three experiments (*P < 0.05; **P < 0.01).

IDO is a negative regulator that induces T cell apoptosis and suppresses anti-tumor immunity. To determine whether GMPF-siIDO could rescue T cells from apoptosis, we collected T cells from spleens of the LLC cancer mice after the treatment with photothermal-immunotherapy by GMPF-siIDO. As shown in Figure 8, the T cell apoptosis was significantly decreased after the photothermal-immunotherapy). The percentages of apoptotic CD8^+^ T cells were significantly decreased (4.2 vs. 22.7%; Figure 8B); similar suppression of apoptotic CD4^+^ T cells was also observed (13.0 vs. 39.9% in Figure 8D). These data suggest that treatment with photothermal-immunotherapy by GMPF-siIDO suppressed apoptosis of T cells in tumor bearing mice.

## Discussion

GNR has obvious localized surface plasmon resonance (LSPR). LSPR refers to the collective oscillation of conduction band electrons caused by the interaction between incident light and the electron cloud on the surface of noble metal nanoparticles, which leads to the absorption and scattering of incident light in the electromagnetic field and produces some unique optical and thermal properties (34, 35). The conversion of absorption light of LSPR into heat is called plasmonic photothermal effects (34, 35). Photothermal therapy (PTT) or plasmonic photothermal therapy (PPTT) refers to the application of the plasma photothermal effects of nanoparticles in the treatment of diseases (10, 36). The plasmonic photothermal effects has achieved selective heating of the local temperature of the tumor to above 50–70°C, which is far igher than the threshold required for vascular injury and cell death (37, 38). The maximum wavelength of resonance absorption of GNR can be tuned to the region of 600–900 nm by changing the axial ratio of rods (12, 39). This near-infrared (NIR) wavelength range is a therapeutic window that can be used in biological applications, so it has been widely used (12, 39, 40). Our study showed that GNR can raise the surrounding temperature to about 74°C under 808 nm laser irradiation, which is far higher than the threshold temperature required to kill tumor cells (28). The main mechanisms of the photothermal effect of GNR to kill tumor cells are necrosis and apoptosis. When the temperature is higher than 50°C, the killing mechanism is mainly necrosis; when the temperature is lower than 50°C, the killing mechanism is mainly apoptosis (28, 29). However, due to the fact that there is no leakage of cell components and no induction of inflammation, apoptosis is considered to be a “clean” way to induce cell death (41). Therefore, we adjusted the concentration of GMPF and the irradiation condition to treat tumors to achieve highly apoptotic percentages *in vitro* and *in vivo* while inducing very low percentages of necrosis (Figures 3, 6).

In addition to its photothermal effects, GNRs possess excellent capacity for carrying siRNA and conjugating targeting adaptors to tumors. To load IDO siRNA and target LLC cells, we utilized a synthesized MUA-PEI-FA coating for the nano-construct GMPF where positively charged PEI absorbs negatively-charged siRNA, in addition, the folic acid (FA) targets FA- receptors that are highly expressed on tumor cells (24–27). Optimal capacities for loading and release of siRNA are essential for the therapy using nano-carriers. There is increasing evidence that the administration of siRNA, either in naked form or wrapped by nanoparticles, distribute mainly to blood-rich organs such as the liver, lungs, kidneys, and spleen, in comparison to tumor tissues where the delivery is lower due to the lack of blood supply (24–27). Therefore, a delivery method that can efficiently carry siRNA to tumor tissues is a highly clinical demand. In this study, we developed a tumor-targeted nanomaterial GMPF that showed high efficiency of delivering siRNA to tumor cells (Figure 4A). Although this method is not tumor specific, we were able to demonstrate that tumor-targeted delivery (e.g., by GMPF) can enhance siRNA delivery to tumor tissues. We note that GMPF is not perfect in its present format and the specific targeting needs to be improved in future studies. In addition, more than 91.5% siRNA can be released effectively from the GMPF-siRNA complex (Figure 2). siIDO was specifically delivered into tumor cells by the nano-construct (Figure 2), and this effectively silenced IDO *in vitro* and *in vivo* (Figures 2, 5). Furthermore, serum is a primary inhibitor for delivering siRNA into cells due to degradation of the siRNA by enzymatic activity (31). In this study, GMPF-siIDO could significantly resist the degradation of siRNA by enzymes in serum for more than 72 h. Therefore, we conclude that GMPF can load, release, and protect siRNA effectively. Moreover, GMPF-siIDO can target LLC tumor tissues and cells and silence IDO molecules efficiently *in vitro* and *in vivo*.

PTT is a promising emerging anti-tumor strategy for not only primary local tumor, but also for distal metastatic tumors *via* “abscopal effects” (15, 16). The therapeutic mechanisms of PTT is mainly involved in hyperthermal killing of tumor cells and upregulating systemic or local immune responses, triggering immune killing by activated TILs. The efficacy of PTT varies depending on the irradiation power, time, and the distance of the irradiation source, in addition to the type of nanomaterials. Theoretically, upregulating the power and/or extending the time of irradiation can allow reaching higher efficiency of PTT, however, this may also damage the normal cells and tissues. Therefore, we optimized the irradiation condition with the power of 2 W/cm^2^ for 5 min, which can maximally protect the normal tissues while killing tumor cells. Unfortunately, PTT-treatment also elevated the expression of IDO, which suppresses tumor cell apoptosis as well as impairs anti-tumor immunity, resulting in the impediment of PTT therapy efficacy (16). Studies have shown that IDO molecules are highly expressed in tumor cells such as LLC (42). Our previous studies have demonstrated that gene silencing of IDO can induce tumor cell apoptosis and sensitize tumor cells to PTT (18, 33), which was also observed in this study (Figure 3). We reported that knocking down IDO enhanced apoptosis of tumor cells through the suppression of NAD^+^, highlighting a novel non-immune mechanism of IDO in tumor growth (33). In this study, the combination of gene silencing of IDO and PTT demonstrated the highest efficacy in anti-tumor therapy (Figure 4). Furthermore, IDO silencing greatly enhanced PTT-inducing apoptosis about 8 folds *in vivo* (Figure 4). Indeed, knockdown of IDO using GMPF-siIDO significantly induced apoptosis of LLC tumor cell *in vitro* and *in vivo* (Figures 3, 6). On the other hand, the most significant feature of PTT treating cancers is inducing tumor cell apoptosis (28, 29). In support of this notion, we observed PTT-induced apoptosis *in vitro* in our previous studies. However, the efficiency of apoptosis is limited for the therapeutic purpose in terms of killing tumor cells *in vivo* (Figures 4B–D). Encouragingly, the use of GMPF-siIDO, which silences checkpoint regulatory molecule IDO in tumors, greatly synergized the tumor cell apoptosis in PTT *in vitro* and especially *in vivo* (Figures 4B–D). These data suggested that IDO is a negative regulator in PTT-induced tumor cell apoptosis; PTT treatment alone is not efficient to induce sufficient tumor cell apoptosis to kill tumor cells in the presence of IDO.

IDO is expressed in immune cells, including dendritic cells. We have previously examined the levels of IDO in DCs and silenced IDO in DCs using siRNA (8). We further demonstrated a therapeutic benefit of gene silencing IDO through a non-specific systemic delivery method using hydro-dynamic injection, which is effective in suppressing tumor growth *in vivo*. *In vivo* application for the treatment of melanoma mice using IDO siRNA showed promising therapeutic effects, including delayed tumor onset and decreased tumor size. Moreover, *in vivo* knockdown of IDO, using siRNA treatment, reinstalled T cell responses and enhanced tumor-specific killing. Therefore, IDO siRNA treatment displayed promising clinical potential through breaking IDO-mediated immune suppression, as well as reinstalling anticancer immunity (20). Additionally, IDO is an essential factor for generating Treg cells. On the other hand, Treg plays a vital role in immune suppression in most cancer patients. We have demonstrated that silencing IDO resulted in enhancing anti-tumor immunity which is associated with the decrease of Treg (8, 18, 43). In agreement with our previously findings, we observed a robust anti-tumor effect (Figure 4) and strong anti-immune response (Figure 7) in this study, when a combination treatment with PPT and siIDO is applied to the tumor-bearing mice. Moreover, IDO as a newly recognized checkpoint molecule exhausts T cells and inhibits proliferation and activation of T cells, and induces regulatory T cells to help tumor cells escape immune surveillance (8). PTT killing tumor cells can activate a systemic immune response (15, 16). However, the presence of immune checkpoint IDO, which is upregulated by apoptotic tumor cells after PTT, may cause significant immune suppression. It has been reported that IDO can induce T cell apoptosis through depleting tryptophan, a critical amino acid for the survival of T cells (44, 45). Therefore, knockdown of IDO can rescue T cells from apoptosis induced by IDO, which has been demonstrated in our previous study (8). We have also demonstrated that tumor cells highly upregulate the expression of IDO which causes apoptosis of both CD4 and CD8 T cells (20). In this study, we observed the increase of infiltrated T cells in the tumors after treatment with siIDO, presumably due to knockdown of IDO, preventing T cells from apoptosis. Moreover, we observed that PTT treatment alone only induced marginal upregulation of anti-tumor immunity. In contrast, when IDO is knocked down, PTT induced robust anti-tumor immunity including enhanced TILs in tumors and increased secretion of anti-tumor inflammatory cytokines (Figure 7). Furthermore, we have previously demonstrated that IDO can suppress anti-tumor immunity through the induction of T cell apoptosis (20). In this study, we further verified that PTT-induced upregulation of IDO also induced T cell apoptosis, whereas knockdown of IDO prevented both CD4^+^ and CD8^+^ T cells from apoptosis (Figure 8). Altogether, these results indicate that gene silencing IDO is capable of synergizing PTT-induced T cell infiltration and cytokine release, suggesting a concert of related mechanisms involved in the anti-tumor immunity after the treatment of GMPF-siIDO. These results also agree with previous studies in which silencing IDO enhanced anti-tumor immunity with increased localization of lymphocytes in tumor tissue (8, 34).

In conclusion, we, for the first time validated the role of IDO as a negative regulator for both PTT-induced tumor cell apoptosis and anti-tumor immunity; IDO is a critical immune checkpoint that impedes the PTT efficiency while combination of gene knockdown of IDO enhances anti-tumor efficacy of PTT. We successfully constructed an immune-stimulatory nano-complex, GMPF-siIDO. GMPF-siIDO can specifically target LLC tumor cells and silence IDO *in vitro* and *in vivo*. Simultaneously, using a novel laser irradiation protocol, GMPF-siIDO exerted synergistic anti-neoplastic effects by inducing tumor cell apoptosis *via* PTT as well as enhancing anti-tumor immunity. Our study provides a novel pipeline design for developing a synergistic clinical treatment method for lung cancer. Therefore, our research developed a new strategy of anti-tumor photothermal-immunotherapy using GMPF-siIDO, which synergizes tumor cell apoptosis *in vivo* through the knockdown of IDO and photothermal mechanisms.

## Data Availability Statement

All datasets generated for this study are included in the article/supplementary material.

## Ethics Statement

The animal study was reviewed and approved by Institutional Animal Care and Use Committee of Nanchang University, China.

## Author Contributions

YZ, KY, and WM planned experiments. YZ, YF, YH, YW, YL, SP, YS, ZW, and YC performed experiments. YZ, KY, WM, RL, QS, NK, and LQ analyzed data. YZ, YF, RJ, KY, and WM wrote the paper. All authors are assured that we met the criteria for authorship, and all reviewed the manuscript. All authors contributed this manuscript.

## Conflict of Interest

The authors declare that the research was conducted in the absence of any commercial or financial relationships that could be construed as a potential conflict of interest.

## Abbreviations

GNR: Gold nanorods
LLC: Lewis lung cancer
FA: Folic acid
IDO: Indoleamine 2,3-dioxygenase
PTT: Photothermal therapy
NIR: near infrared radiation
TEM: Transmission electron microscope
NMR: Nuclear magnetic resonance
CTAB: Cetyltrimethylammonium bromide
MUA: Mercaptoundecanoic acid
PEI: Poly ethylene imine
GMPF: GNR-MUA-PEI-FA construct
siRNA: small interfering RNA
LSPR: Localized surface plasmon resonance.
